# Mutual Information and Quantum Coherence in Minimum Error Discrimination of *N* Pure Equidistant Quantum States

**DOI:** 10.3390/e27080863

**Published:** 2025-08-14

**Authors:** Omar Jiménez

**Affiliations:** Centro Multidisciplinario de Física, Vicerrectoría de Investigación, Universidad Mayor, Santiago 8580745, Chile; omar.jimenez@umayor.cl

**Keywords:** mutual information, quantum coherence, quantum state discrimination, equidistant states

## Abstract

We study the quantum state discrimination problem under the minimum error (ME) strategy for a set of *N* pure equidistant states. These states are characterized by the property that the inner product between any pair of states is given by a unique complex number *S*. We provide the explicit form of the states and analyze their main structural properties. The optimal success probability for ME discrimination is evaluated as a function of the number of states, as well as the modulus and phase of the inner product *S*. Furthermore, we propose an experimental scheme for implementing the ME discrimination of equidistant states. We also investigate the quantum coherence consumed in the implementation of the minimum error discrimination of the equidistant states, which has an established operational interpretation as cryptographic randomness gain. As an application, we propose a quantum communication protocol in which Alice prepares and sends one of the equidistant states, while Bob applies the minimum error discrimination to extract the classical information encoded in the state. Finally, we discuss the optimal conditions under which the protocol achieves an optimal balance of classical correlations and quantum coherence, thereby ensuring effective information transfer and cryptographic security.

## 1. Introduction

One of the main applications of quantum information theory is the development of quantum communication protocols [1,2]. In such protocols, two legitimate parties, commonly referred to as Alice and Bob, aim to share information securely. Meanwhile, an adversary, known as Eve, attempts to intercept the information without being detected. To establish communication, Alice and Bob agree on a set of quantum states, which are typically chosen to be nonorthogonal. A key property of nonorthogonal quantum states is that they cannot be perfectly distinguishable by any quantum measurement [3,4]. This inherent indistinguishability underlies the security of quantum communication protocols, such as the well-known BB84 scheme [5]. In a typical communication protocol, Alice prepares and transmits classical information encoded in a set of nonorthogonal quantum states. At the distant end of the communication channel, Bob receives the quantum state and performs a measurement to extract the information sent by Alice. The specific quantum measurement employed by Bob is chosen to optimize a predefined figure of merit. For example, he may implement a measurement that minimizes the probability of error in identifying the transmitted states (ME) [6,7,8] or one that allows for the extraction of the accessible information (MI) [9,10,11], which quantifies the maximal classical correlation that can be established between the legitimate parties. In general, these correspond to two distinct optimization problems [12]. However, when the set consists of two pure nonorthogonal states prepared with arbitrary a priori probabilities, it is known that ME and MI measurements coincide [4,11,12,13,14,15]. The optimization problem consists of finding the optimal set of quantum measurement operators. For minimum error discrimination, the necessary and sufficient conditions that the optimal measurement operators must satisfy are well established [6,7]. In contrast, for the accessible information, only necessary conditions are known [6,16,17]. Analytical solutions for the ME strategy are available for specific classes of quantum states [6,7,8,17,18,19,20,21]. On the other hand, the MI optimization problem is significantly more challenging, and exact solutions are known only for a few cases [22,23,24]. Nevertheless, there exists lower and upper bounds for the accessible information [16,25,26], and several results have been reported regarding the number of measurement operators required to implement the MI strategy [10,11]. Minimum error discrimination plays a key role in various quantum information processing tasks, including quantum teleportation [27,28], entanglement swapping [29,30], quantum cryptography [31], and dense coding [32], among others. Furthermore, the ME of nonorthogonal states has been successfully demonstrated in several experimental settings [12,33,34,35]. On the other hand, the accessible information strategy finds application in quantum cryptography [16], and its experimental implementation has been reported in specific scenarios [12].

In this work, we study the minimum error discrimination of a set of *N* pure, nonorthogonal equidistant quantum states, each prepared with equal a priori probability. We derive the optimum measurement operators and the corresponding success probability for ME, and we also propose an experimental scheme for its implementation. Moreover, we evaluate the quantum coherence, involved in applying the ME strategy to the equidistant states, which has the operational interpretation as a cryptographic randomness gain. We then determine the classical correlations shared between Alice and Bob when the ME strategy is employed. Finally, we study the relationship between classical correlations and quantum coherence within the ME protocol. Interestingly, our results reveal a fundamental trade-off: greater classical information sharing between Alice and Bob corresponds to reduced randomness generation, and vice versa. Our analysis is carried out in scenarios where they share either a bipartite separable state or operate within a prepare-and-measure framework that does not involve pre-shared entanglement between the sender and receiver. Consequently, our scheme does not exhibit memory effects, such as those considered in Ref. [36], nor does it involve uncertainty trade-offs arising from measurements of incompatible observables, as studied in Refs. [37,38].

This article is organized as follows: In Section 2, we introduce and describe the set of *N* pure, nonorthogonal equidistant quantum states. In Section 3, we derive the optimal measurement operators and the corresponding success probability for the minimum error discrimination of these states, together with a proposal for their experimental implementation. In Section 4, we focus on the analysis of quantum coherence of the set of equidistant states. In Section 5, we describe the initial and final global states of the composite system shared by Alice and Bob, resulting from the minimum error (ME) measurement performed by Bob. In Section 6, we study the classical correlations shared between Alice and Bob when ME is implemented by Bob. Moreover, we examine the trade-off between Bob’s information gain and the quantum coherence consumed in the process. Finally, in Section 7, we summarize our findings and present concluding remarks.

## 2. Equidistant States

Let us consider a set of *N* pure, nonorthogonal quantum states denoted by $\left\{|\psi_{j}\rangle \right\}$ with j=0,…,N−1, satisfying the following property:

(1) $$\langle \psi_{j}|\psi_{j^{\prime }}\rangle =S=\left|S\right|e^{i\theta },\;\forall \;j>j^{\prime },$$

that is, the inner product between any two states in the set depends solely on a single complex number *S* or, equivalently, on two real parameters: its modulus |S| and its phase θ. Due to this property, the set is referred to as equidistant [39,40,41]. For such a set of states, the modulus |S| is constrained to lie within the interval $\left|S|\in [0,|S_{\theta }|\right]$, where $|S_{\theta }|$ is a function of the phase θ and the number *N* of states in the set, given by

(2) $$|S_{\theta }|=\frac{\sin\left(\frac{\pi -\theta }{N}\right)}{\sin\left(\theta +\frac{\pi -\theta }{N}\right)}.$$

The explicit form of the set of *N* pure, nonorthogonal equidistant states is given in [40] as

(3) $$|\psi_{j}\rangle =\sum_{k=0}^{N-1}C_{k}\omega_{k}^{j}|k\rangle ,\;\mathrm{for}\;j=0,1,\ldots ,N-1,$$

where *N* is fixed, and all the real coefficients $C_{k}$ in Equation (3) depend only on the modulus |S| and the phase θ of the inner product *S*. These coefficients are given by

(4) $$C_{k}=\sqrt{\frac{1}{N}\left[1-\left|S\right|\frac{\sin\left(\theta +\frac{k\pi -\theta }{N}\right)}{\sin\left(\frac{k\pi -\theta }{N}\right)}\right]},\;\mathrm{for}\;k=0,1,\ldots ,N-1.$$

Given the symmetry of the equidistant states, we restrict the phase θ of the inner product *S* to the interval [0,π]. Within this interval, the coefficients exhibit an ordering property, namely

(5) $$C_{1}\leq C_{2}\leq C_{3}\leq \ldots \leq C_{0},$$

and the phases $\omega_{k}$ in Equation (3) are defined as

(6) $$\omega_{k}=e^{\frac{2i}{N}\left(k\pi -\theta \right)},\;\mathrm{for}\;k=0,1,\ldots ,N-1.$$

**Figure 1:**
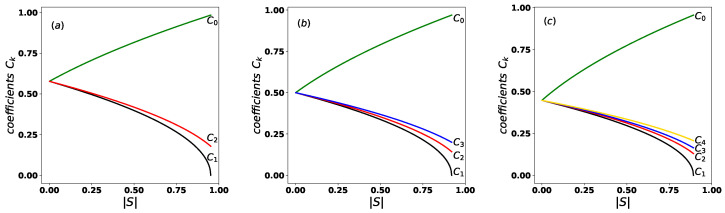
Ordering property of the coefficients $C_{k}$ from Equation (5) as a function of |S|, shown for (**a**) N=3, (**b**) N=4, and (**c**) N=5, with θ=π/36.

Figure 1 illustrates the ordering of the coefficients $C_{k}$, as defined in Equation (5), as a function of |S|, for (a) N=3, (b) N=4, and (c) N=5, with θ=π/36. As shown in all cases, the maximum and minimum values of the coefficients correspond to $C_{0}$ and $C_{1}$, respectively. Moreover, when the states are orthogonal (|S|=0), all coefficients become equal and take the value $1/\sqrt{N}$, where *N* is the number of states in the set. Given the coefficients $C_{k}$ defined in Equation (4), the equidistant states in Equation (3) are properly normalized, that is,

(7) $$\sum_{k=0}^{N-1}C_{k}^{2}=1.$$

**Figure 2:**
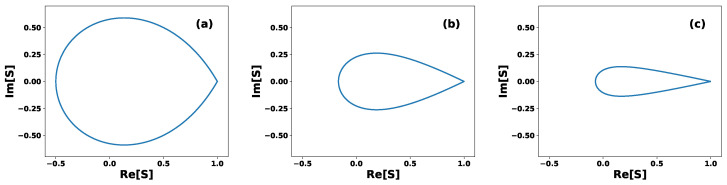
Polar graph of the inner product *S*. The blue line indicates the boundary $\left|S|=|S_{\theta }\right|$, where the set of equidistant states becomes linearly dependent. Each point inside the bounded region corresponds to a specific value of *S* and represents a set of *N* linearly independent equidistant states: (**a**) *N* = 3, (**b**) *N* = 7, and (**c**) *N* = 15.

Figure 2 shows, in polar coordinates, the possible values of the inner product *S* for sets of pure equidistant states, with (a) *N* = 3, (b) *N* = 7, and (c) *N* = 15. For a given phase θ, the modulus of the inner product *S* is constrained to lie within the interval $\left|S|\in [0,|S_{\theta }|\right]$. The blue line in Figure 2 corresponds to the case $\left|S|=|S_{\theta }\right|$, for which the set of equidistant states becomes linearly dependent. For instance, when θ=0, the condition $|S|=|S_{\theta }|=1$ implies that all *N* states are all identical to |0〉. In this case, the states span a one-dimensional Hilbert space and are therefore linearly dependent. For any other value of θ, the condition $\left|S|=|S_{\theta }\right|$ defines a set of *N* equidistant states that are linearly dependent and span an (N−1)-dimensional Hilbert space. On the other hand, the set of pure equidistant states is linearly independent when the modulus of the inner product lies within the region bounded by the blue line, i.e., for $\left|S|\in [0,|S_{\theta }|\right)$. In this case, the states span an *N*-dimensional Hilbert space. Figure 2 illustrates how the range of possible values for |S| decreases as the number of states *N* increases. This implies that, as *N* increases, the phase θ of the inner product becomes progressively less relevant, ultimately leaving only the case θ=0 in the limit of large *N*. Another important property of the set of equidistant states is that there exists a unitary transformation *U* that generates the entire set from a single state, namely

(8) $$|\psi_{j}\rangle =U^{j}|\psi_{0}\rangle ,\;\mathrm{with}\;j=0,1,2,\ldots ,N-1,$$

where

(9) $$U=\sum_{k=0}^{N-1}\omega_{k}|k\rangle \langle k|,$$

with $\omega_{k}$ defined in Equation (6), and $|\psi_{0}\rangle$ is referred to as the fiducial state. Applying the unitary transformation *N* times yields

(10) $$U^{N}=\sum_{k=0}^{N-1}\omega_{k}^{N}\left|k\rangle \langle k\right|=\sum_{k=0}^{N-1}e^{2i(k\pi -\theta )}\left|k\rangle \langle k\right|=e^{-2\theta i}1_{N}.$$

Under the condition $U^{N}=1_{N}$, the set of equidistant states is also symmetric. This occurs when θ=0 or θ=π. In the following, we provide a more detailed description of these two particular sets of states.

For θ=0, the inner product is a real and positive number, with S=|S| and |S|∈[0,1]. In this case, the fiducial state takes the form

(11) $$|\psi_{0}\rangle =C_{0}|0\rangle +C_{1}\sum_{k=1}^{N-1}|k\rangle ,$$

where the coefficients are given by $C_{1}=\sqrt{(1-|S|)/N}$ and $C_{0}=\sqrt{C_{1}^{2}+\left|S\right|}$. The set of *N* equidistant states is linearly independent (dependent), and spans an *N*-dimensional (1-dimensional) Hilbert space, if |S|∈[0,1)(|S|=1), respectively.

For θ=π, the inner product is real and negative, with S=−|S| and $\left|S|\in [0,\frac{1}{N-1}\right]$. The fiducial state in this case takes the form

(12) $$|\psi_{0}\rangle =C_{1}|1\rangle +C_{0}\sum_{k=0,k\neq 1}^{N-1}|k\rangle ,$$

with coefficients $C_{1}=\sqrt{(1-(N-1)|S|)/N}$ and $C_{0}=\sqrt{C_{1}^{2}+\left|S\right|}$. Here, the set of *N* equidistant states is linearly independent (dependent), and spans an *N*-dimensional (*N* − 1 dimensional) Hilbert space, if $\left|S\right|\in \left[0,\frac{1}{N-1}\right)\;(|S|=\frac{1}{N-1})$, respectively.

To highlight the differences between a set of equidistant states and a set of symmetric states, we first summarize the main properties of symmetric states [3,4,17]. In general, a set of *N* symmetric states $\{|\phi_{j}\rangle {\}}_{j=0}^{N-1}$, spanning an *N*-dimensional Hilbert space, can be generated from a single fiducial state $|\phi_{0}\rangle$, which has the form

(13) $$|\phi_{0}\rangle =\sum_{k=0}^{N-1}C_{k}|k\rangle .$$

The remaining states in the set are obtained by the repeated application of a unitary operator *U* on the fiducial state:

(14) $$|\phi_{j}\rangle =U^{j}|\phi_{0}\rangle ,\;\mathrm{with}\;j=0,1,2,\ldots ,N-1,$$

where *U* is defined by

(15) $$U=\sum_{k=0}^{N-1}e^{\frac{2\pi ik}{N}}|k\rangle \langle k|.$$

This unitary operator satisfies the cyclic property $U^{N}=1_{N}$, where $1_{N}$ denotes the identity operator in the *N*-dimensional Hilbert space. The coefficients $C_{k}$ in the fiducial state are arbitrary complex numbers, subject only to the normalization condition:

(16) $$\sum_{k=0}^{N-1}{\left|C_{k}\right|}^{2}=1.$$

A set of *N* symmetric states generally contains both complex and real inner products among its elements. For example, in the case N=4, the inner products between the fiducial state and the other states are given by

(17) $$\begin{array}{ccc} \langle \phi_{0}|\phi_{1}\rangle & = & {\langle \phi_{0}|\phi_{3}\rangle }^{*}=|C_{0}{|}^{2}+i|C_{1}{|}^{2}-|C_{2}{|}^{2}-i{\left|C_{3}\right|}^{2}, \end{array}$$

(18) $$\begin{array}{ccc} \langle \phi_{0}|\phi_{2}\rangle & = & |C_{0}{|}^{2}-|C_{1}{|}^{2}+|C_{2}{|}^{2}-{\left|C_{3}\right|}^{2}. \end{array}$$

Hence, the structure and mutual overlaps of symmetric states depend on N−1 free parameters (the *N* modulus of the *N* coefficients minus the normalization condition), making their analytical treatment increasingly complex as *N* increases.

In contrast, for a set of equidistant states, the structure is far more constrained. The states are generated by a specific unitary transformation *U* defined in Equation (9), and their coefficients are fixed by Equation (4). Most importantly, the inner product between any pair of distinct equidistant states is always the same and given by a single complex number *S*, as defined in Equation (1). This implies that the entire set of equidistant states is fully characterized by only two real parameters: the modulus |S| and the phase θ of the inner product *S*. Remarkably, this remains true regardless of the number *N* of states in the set.

As a result, the equidistant states offer a simpler and more tractable structure for analytical and numerical analyses, especially in high-dimensional Hilbert spaces. Furthermore, their uniform overlap properties make them useful in various quantum information applications, including minimum error state discrimination, quantum communication protocols, and coherence-based resource analyses.

## 3. Minimum Error Discrimination

Having defined the set of *N* pure nonorthogonal equidistant states $\{|\psi_{j}\rangle {\}}_{j=0}^{N-1}$ in [39] and described it in detail in [41], we now study its quantum state discrimination under the minimum error strategy. To this end, we assume that each state $|\psi_{j}\rangle$ is prepared with equal a priori probability, i.e., $\eta_{j}=1/N$ for j=0,1,…,N−1.

In general, the necessary and sufficient conditions for optimum discrimination with minimum error among *N* density matrices $\rho_{k}$, each prepared with arbitrary a priori probabilities $\eta_{k}$, were found by Holevo [6] and Yuen [7]. These conditions are given by

(19) $$\begin{array}{c} \Pi_{k}\left(\eta_{k}\rho_{k}-\eta_{j}\rho_{j}\right)\Pi_{j}=0,\;\forall \;j,k, \end{array}$$

(20) $$\begin{array}{c} \left(\sum_{k}\eta_{k}\Pi_{k}\rho_{k}\right)-\eta_{j}\rho_{j}\geq 0,\;\forall \;j, \end{array}$$

where $\Pi_{k}$ are the detection operators to be determined for the optimum discrimination of the state $\rho_{k}$. In the particular case of a set of *N* pure nonorthogonal equidistant states prepared with equal a priori probability, the above conditions are satisfied when the detection operators are given by

(21) $$\Pi_{k}=|u_{k}\rangle \langle u_{k}|,\;\mathrm{for}\;k=0,1,\ldots ,N-1,$$

where the states $|u_{k}\rangle$ are defined as

(22) $$|u_{k}\rangle =\frac{1}{\sqrt{N}}\sum_{j=0}^{N-1}e^{2\pi ijk/N}|j\rangle ,\;\mathrm{for}\;k=0,1,\ldots ,N-1,$$

and form an orthonormal basis generated by the discrete Fourier transform *F* acting on the *N*-dimensional Hilbert space, i.e., $|u_{k}\rangle =F|k\rangle$, with

(23) $$F=\frac{1}{\sqrt{N}}\sum_{j,m=0}^{N-1}e^{2\pi ijm/N}|j\rangle \langle m|.$$

Thus, there is a one-to-one correspondence between each orthonormal states $|u_{k}\rangle$ and one state from the computational basis |k〉. Moreover, the detection operators $\Pi_{k}$, defined in Equation (21), form a complete set in the *N*-dimensional Hilbert space:

(24) $$\sum_{k=0}^{N-1}\Pi_{k}=1_{N}.$$

In general, the success probability $P_{s}$ for the ME of *N* quantum states $\rho_{k}$, each prepared with a priori probability $\eta_{k}$, is given by [3,4]

(25) $$P_{s}=\sum_{k=0}^{N-1}\eta_{k}tr\left(\Pi_{k}\rho_{k}\right).$$

For the case of *N* pure nonorthogonal equidistant states, $\rho_{k}=|\psi_{k}\rangle \langle \psi_{k}|$, prepared with equal a priori probabilities ($\eta_{k}=1/N$), the optimum success probability simplifies to

(26) $$P_{s}={\left|\langle u_{0}|\psi_{0}\rangle \right|}^{2},$$

as a consequence of the symmetry of the state set and the structure of the detector operators $\Pi_{k}=|u_{k}\rangle \langle u_{k}|$, defined in Equation (21). The corresponding minimum error probability is then

(27) $$P_{e}=1-P_{s}.$$

Therefore, the optimum success probability in ME of *N* pure nonorthogonal equidistant states with equal a priori probabilities is given by

(28) $$P_{s}=\frac{1}{N}{\left(\sum_{k=0}^{N-1}C_{k}\right)}^{2},$$

where the coefficients $C_{k}$ correspond to those of the fiducial state $|\psi_{0}\rangle$. The success probability given by Equation (28) is similar to that obtained for the ME of *N* symmetric states prepared with equal a priori probabilities [17,42,43]. We notice here that using detector operators different from $\Pi_{k}=|u_{k}\rangle \langle u_{k}|$ results in a higher probability of error. The worst case scenario occurs when the state is guessed randomly, which is equivalent to using the detector operators of the form $\Pi_{k}^{\prime }=\left|k\rangle \langle k\right|$, that is, performing a direct measurement of the equidistant states $|\psi_{j}\rangle$ in the computational basis ${\{|k\rangle \}}_{k=0}^{N-1}$. In such a case, the success probability is $P_{s}=1/N$, independently of the form of the states. A similar result is obtained even when using the optimum detector operators $\Pi_{k}=|u_{k}\rangle \langle u_{k}|$, if the inner product between the states satisfies |S|=1. This corresponds to the situation in which all the states are identical, i.e., $|\psi_{j}\rangle =|0\rangle$ for j=0,1,…,N−1. Since the states are completely indistinguishable in this scenario, the optimal success probability again reduces to $P_{s}=1/N$.

**Figure 3:**
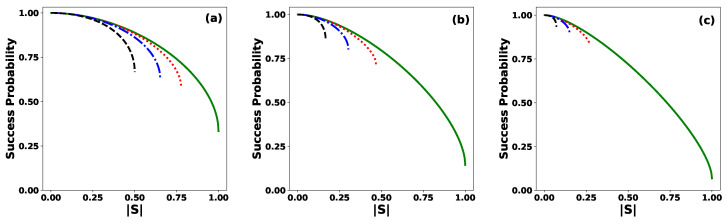
Success probability $P_{s}$ for the ME of equidistant states as a function of the modulus |S| of the inner product for the values θ=0 (solid green line), θ=π/6 (dotted red line), θ=π/3 (dashed–dotted blue line), and θ=π (dashed black line), for (**a**) N=3, (**b**) N=7, and (**c**) N=15.

Figure 3 shows the optimal success probability $P_{s}$, given by Equation (28), in the discrimination by the ME of *N* equidistant states as a function of |S|, for several values of θ, and for (a) N=3, (b) N=7, and (c) N=15. When the states are orthogonal, |S|=0, the maximum success probability $P_{s}=1$ is achieved. This shows the well-known result that orthogonal states can be perfectly discriminated deterministically and without error. In all other cases, the discrimination involves some error, but this strategy minimizes the error probability. For any fixed value of *N* and θ, the success probability $P_{s}$ decreases as |S| increases, reaching its minimum value when the states become linearly dependent, that is, when $\left|S|=|S_{\theta }\right|$. On the other hand, if the number of states *N* and the modulus |S| are fixed (within the allowed range), the success probability $P_{s}$ decreases as the phase θ increases. This implies that it is more likely to correctly discriminate a set of linearly independent states than a set of linearly dependent ones. Moreover, as the number of states *N* increases, the only relevant case becomes θ=0. This is because, for θ≠0, the allowed values of |S| become increasingly close to zero, implying that the success probability under the ME strategy tends to one.

The ME strategy can be interpreted as a quantum communication scenario. On one side, Alice prepares and sends a quantum state $|\psi_{j}\rangle$, chosen from the set of equidistant states. On the other side, Bob, located at a distant location, receives the state and applies quantum state discrimination to retrieve the classical information encoded in the state. To implement ME, Bob must first apply a unitary transformation to the received states that he received $|\psi_{j}\rangle$. For the set of equidistant states considered here, the appropriate transformation is the discrete inverse Fourier transform $F^{-1}$. This operation transforms the equidistant states according to

(29) $$F^{-1}|\psi_{j}\rangle =\sum_{n=0}^{N-1}C_{n}\omega_{n}^{j}|u_{N-n}\rangle ,\;\mathrm{for}\;j=0,1,\ldots ,N-1,$$

where

(30) $$|u_{N-n}\rangle =\frac{1}{\sqrt{N}}\sum_{k=0}^{N-1}e^{-2\pi ikn/N}|k\rangle ,\;\mathrm{for}\;n=0,1,\ldots ,N-1,$$

and thus, the evolution of the equidistant states under the $F^{-1}$ transformation is given by

(31) $$F^{-1}|\psi_{j}\rangle =\frac{e^{-2i\theta j/N}}{\sqrt{N}}\sum_{k,n=0}^{N-1}C_{n}e^{2\pi in(j-k)/N}|k\rangle ,\;\mathrm{for}\;j=0,1,\ldots ,N-1.$$

We assume that the subtraction N−n in Equations (29) and (30) is understood to be modulo *N*. After applying the discrete inverse Fourier transform $F^{-1}$, Bob completes the ME by performing a projective measurement on the transformed states $|{\hat{\psi }}_{j}\rangle =F^{-1}|\psi_{j}\rangle$ in the computational basis {|k〉}. For instance, if Alice prepares and sends the state $|\psi_{1}\rangle$, Bob applies the transformation and obtains $|{\hat{\psi }}_{1}\rangle =F^{-1}|\psi_{1}\rangle$. He then performs a projective measurement in the basis {|k〉}, which yields one of *N* possible outcomes. If the outcome is |1〉, the state $|\psi_{1}\rangle$ has been correctly identified, and the discrimination is successful. The corresponding success probability is given by $P_{s}={\left|\langle 1|F^{-1}|\psi_{1}\rangle \right|}^{2}={\left|\langle 1|{\hat{\psi }}_{1}\rangle \right|}^{2}$. Conversely, if the outcome is |k〉 with k≠1, an error occurs in the discrimination of $|\psi_{1}\rangle$. However, The ME strategy guarantees that this error occurs with the lowest possible probability among all quantum discrimination strategies.

**Figure 4:**
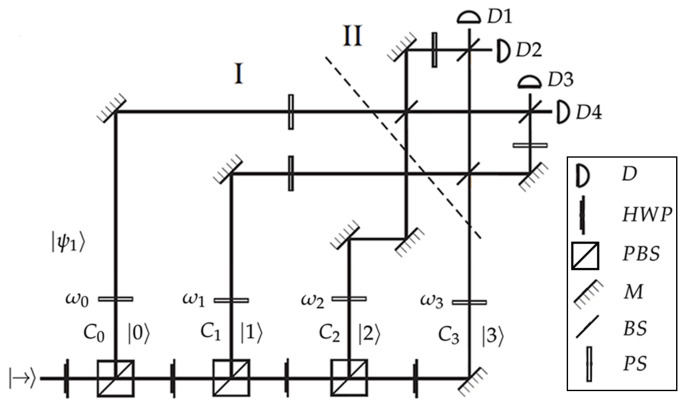
Experimental proposal for ME of four equidistant states: (I) State preparation: Alice sends one of the equidistant states, e.g., $|\psi_{1}\rangle$. (II) Detection stage: Bob applies the inverse discrete Fourier transform $F^{-1}$ in a four-dimensional Hilbert space and performs a projective measurement in the computational basis {|k〉}. HWP, half-wave plate; PBS, polarizing beam splitter; PS, phase shifter; BS, beam splitter; M, mirror; D, detector.

A proposal for the experimental implementation of an ME of N=4 pure equidistant states is shown in Figure 4. This scheme is similar to previously reported setups for the discrimination of symmetric states [43,44]. The first stage corresponds to the state preparation. Alice prepares and sends one of the equidistant states, say $|\psi_{1}\rangle$. For this purpose, a single photon with horizontal polarization |→〉 enters the experimental setup. Alice can prepare any of the equidistant states by adjusting the phases $\omega_{k}$ in each propagation path of the photon |k〉, and by setting the appropriate rotation angles in the polarizing beam splitters to generate the desired amplitudes $C_{k}$ for each path. The prepared state then propagates through the channel. Upon receiving the state $|\psi_{1}\rangle$, Bob implements the inverse of the discrete Fourier transform $F^{-1}$ in a four-dimensional Hilbert space. Finally, he performs a projective measurement in the computational basis {|k〉}. The initial single photon, after propagating through the discrimination setup, will be detected by one of the four detectors shown in Figure 4.

## 4. Quantum Coherence

As previously mentioned, in a quantum communication scenario, Alice prepares and sends to Bob one of the equidistant states $|\psi_{j}\rangle$, each with equal a priori probability $\eta_{j}=1/N$. Bob aims to extract the encoded information by performing a quantum measurement on the received state. Therefore, the ensemble of possible states received by Bob is described by the density matrix $\rho_{B}$, which is given by

(32) $$\rho_{B}=\frac{1}{N}\sum_{j=0}^{N-1}|\psi_{j}\rangle_{B}\langle \psi_{j}|.$$

Due to the symmetry of the equidistant states, this density matrix takes the simplified diagonal form

(33) $$\rho_{B}=\sum_{k=0}^{N-1}C_{k}^{2}{|k\rangle }_{B}\langle k|,$$

which is a diagonal state in the computational basis ${\{|k\rangle }_{B}\}$. Quantum coherence is associated with the ability of a quantum system to exhibit interference effects [45]. Such interference arises when the system’s density matrix possesses non zero off-diagonal elements in a given basis. Accordingly, the state $\rho_{B}$, in Equation (33), exhibits no quantum coherence with respect to the basis ${\{|k\rangle }_{B}\}$, as it is diagonal in that basis. A canonical example of a maximally coherent state in a *d*-dimensional Hilbert space is given by [46,47]

(34) $$|\psi \rangle =\frac{1}{\sqrt{d}}\sum_{k=0}^{d-1}|k\rangle ,$$

which contains $\log_{2}d$ bits of coherence, also referred to as cobit [46], relative to the computational basis {|k〉}. Quantum coherence has been formally established as a fundamental resource for the implementation of quantum protocols, and it is consumed during the execution of such protocols [46,48,49]. Several measures have been proposed to quantify quantum coherence, including the coherence cost, the relative entropy of coherence, and the $\ell_{1}$ norm of coherence, among others [46]. To quantify the quantum coherence of the state $\rho_{B}$ with respect to a given projective measurement Π, we employ the relative entropy of coherence [46,47], defined as

(35) $$C\left(\rho_{B},\Pi \right)=H\left(p_{i}\right)-S\left(\rho_{B}\right),$$

where $H(p_{i})$ is the Shannon entropy associated with the measurement outcome probabilities $\left\{p_{i}\right\}$, given by

(36) $$H\left(p_{i}\right)=-\sum_{i=0}^{N-1}p_{i}\log_{2}p_{i},$$

and where

(37) $$p_{i}=tr\left(\rho_{B}\Pi_{i}\right),$$

with $\Pi ={\left\{\Pi_{i}\right\}}_{i=0}^{N-1}$ being the projective measurement performed by Bob, and S(ρ) is the von Neumann entropy, which is

(38) $$S\left(\rho \right)=-\sum_{k=0}^{N-1}\lambda_{k}\log_{2}\lambda_{k},$$

where $\lambda_{k}$ are the eingenvalues of the density matrix ρ. For the set of equidistant states, the von Neumann entropy of $\rho_{B}$ is given by

(39) $$S\left(\rho_{B}\right)=-\sum_{k=0}^{N-1}C_{k}^{2}\log_{2}C_{k}^{2},$$

which depends solely on the coefficients $C_{k}$ and is independent of the quantum measurement Π applied by Bob. As shown in Equation (35), the quantum coherence depends both on the state $\rho_{B}$ and on the quantum measurement Π, which, in this case, corresponds to ME. Then, the probability distribution $\left\{p_{i}\right\}$ is given by

(40) $$p_{i}=tr\left(\rho_{B}\Pi_{i}\right)=\frac{1}{N},\;\mathrm{for}\;i=0,1,\ldots ,N-1.$$

Therefore, the relative entropy of coherence when Bob implements ME is given by

(41) $$C\left(\rho_{B},\Pi \right)=\log_{2}N+\sum_{k=0}^{N-1}C_{k}^{2}\log_{2}C_{k}^{2},$$

which corresponds to the maximum coherence attainable from any projective measurement Π on the state $\rho_{B}$. This result holds because the Shannon entropy $H(p_{i})$ reaches its maximum value, $H\left(p_{i}\right)=\log_{2}N$, when the measurement is performed using the projectors $\Pi_{k}$ defined in Equation (21). Thus, among all possible projective measurements, the ME strategy maximizes the quantum coherence of $\rho_{B}$.

**Figure 5:**
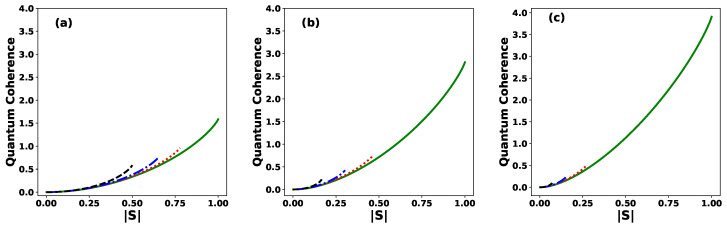
Quantum coherence of the initial state $\rho_{B}$, given in Equation (33), as a function of the modulus |S| of the inner product, for different values of the phase: θ=0 (solid green line), θ=π/6 (dotted red line), θ=π/3 (dashed–dotted blue line), and θ=π (dashed black line) for (**a**) N=3, (**b**) N=7, and (**c**) N=15.

Quantum coherence plays a fundamental operational role in the context of cryptographic randomness gain [50,51]. In particular, when an eavesdropper (Eve) is present in the communication channel, higher values of quantum coherence make it more difficult for her to extract information. For instance, if the set of quantum states is orthogonal, |S|=0, the quantum coherence vanishes, and Eve can obtain complete information by performing a projective measurement without disturbing Bob’s state $\rho_{B}$. At the opposite extreme, when all the states are identical, i.e., $|\psi_{j}\rangle =|0\rangle$, for all *j*, the state is pure, $\rho_{B}={|0\rangle }_{B}\langle 0|$, and the quantum coherence reaches its maximum value of $\log_{2}N$, where *N* the number of states in the ensemble. In this scenario, Eve cannot distinguish between the states and is left with no better strategy than randomly guessing the state sent by Alice. Figure 5 shows the quantum coherence of $\rho_{B}$ when the ME of *N* pure equidistant states is implemented as a function of |S| for various values of the phase θ for (a) N=3, (b) N=7, and (c) N=15. The behavior of quantum coherence $C(\rho_{B},\Pi )$ differs notably from that of the success probability $P_{s}$. For any given value of θ and *N*, coherence reaches its minimum (zero) when the states are orthogonal |S|=0, and its maximum ($\log_{2}N$) when the states are linearly dependent with $|S_{\theta =0}|\;=1$. For a fixed number of states *N*, within the allowed range of |S|, increasing the value of the phase θ from θ=0 to θ=π results in an increase in quantum coherence. This indicates that, for a given *N*, a more linearly dependent set of states exhibit greater coherence than a linearly independent one. Furthermore, for any fixed θ≠0, increasing the number of states *N* leads to a rapid decrease in coherence, which tends to zero as *N* becomes sufficiently large.

## 5. Channel Without Entanglement

In the ME scheme, Alice prepares a single copy of a quantum system in the state $|\psi_{j}\rangle$ and sends it to Bob with an a priori probability $\eta_{j}=1/N$. We assume that, initially, Alice and Bob share a separable quantum state $\rho_{AB}$ of the form

(42) $$\rho_{AB}=\frac{1}{N}\sum_{j=0}^{N-1}{|j\rangle }_{A}\langle j|⊗|\psi_{j}\rangle_{B}\langle \psi_{j}|,$$

where ${\{|j\rangle }_{A}{\}}_{j=0}^{N-1}$ forms an orthonormal base for Alice’s *N*-dimensional quantum system, and ${\{|\psi_{j}\rangle }_{B}{\}}_{j=0}^{N-1}$ are the pure equidistant states that Bob receives. Therefore, Alice and Bob share quantum and classical correlations encoded in the joint state $\rho_{AB}$ defined in Equation (42). The initial state $\rho_{A}$ of Alice’s quantum system, that is, prior to the application of any transformation or measurement, is obtained by $\rho_{A}={\mathrm{tr}}_{B}\left(\rho_{AB}\right)$, where

(43) $$\rho_{A}=\frac{1}{N}\sum_{j=0}^{N-1}{|j\rangle }_{A}\langle j|.$$

In a similar form, the initial state of Bob’s quantum system can be obtained by tracing out Alice’s subsystem from the global state, i.e., $\rho_{B}={\mathrm{tr}}_{A}\left(\rho_{AB}\right)$, where

(44) $$\rho_{B}=\frac{1}{N}\sum_{j=0}^{N-1}|\psi_{j}\rangle_{B}\langle \psi_{j}|.$$

Once Bob receives a single copy of the quantum system prepared in one of the equidistant state $|\psi_{j}\rangle_{B}$, he implements the ME strategy. For that purpose, Bob first applies the unitary transformation $F^{-1}$ to his quantum system, thereby transforming the global state $\rho_{AB}$ into a new state ${\hat{\rho }}_{AB}$, given by

(45) $${\hat{\rho }}_{AB}=\frac{1}{N}\sum_{j=0}^{N-1}{|j\rangle }_{A}\langle j|⊗|\hat{\psi_{j}}\rangle_{B}\langle \hat{\psi_{j}}|,$$

where ${|\hat{\psi_{j}}\rangle }_{B}=F^{-1}{|\psi_{j}\rangle }_{B}$. The unitary operation $F^{-1}$, defined in Equation (23) and applied by Bob, is a reversible process [52]. Therefore, it does not change the quantum correlations between Alice and Bob initially encoded in the global state $\rho_{AB}$. After this transformation, Bob performs a measurement on his subsystem, which yields *N* possible outcomes and, correspondingly, *N* conditional post-measurement states $\rho_{A|B}^{k}$ for Alice’s subsystem. Since Bob’s measurement projects the state $|{\hat{\psi }}_{j}\rangle_{B}$ onto one of the orthonormal basis states ${|k\rangle }_{B}$, the resulting conditional state for Alice is given by

(46) $$\rho_{A|B}^{k}=\sum_{j=0}^{N-1}P_{kj}|j\rangle \langle j|,\;\mathrm{for}\;k=0,1,\ldots ,N-1,$$

where

(47) $$P_{kj}=|\langle k|{\hat{\psi }}_{j}\rangle {|}^{2}={\left|\langle u_{k}|\psi_{j}\rangle \right|}^{2}=\frac{1}{N}{\left|\sum_{n=0}^{N-1}C_{n}\;e^{2\pi i(k-j)n/N}\right|}^{2},$$

where $P_{kk}=P_{s}={\left|\langle k|{\hat{\psi }}_{k}\rangle \right|}^{2}$ is the success probability in ME for each k=0,1,…,N−1. The final average joint state between Alice and Bob, denoted by $\rho_{AB}^{\prime }$, after Bob performs his measurement in the basis ${\{|k\rangle }_{B}\}$, takes the following form:

(48) $$\rho_{AB}^{\prime }=\frac{1}{N}\sum_{k=0}^{N-1}\rho_{A|B}^{k}⊗{|k\rangle }_{B}\langle k|.$$

Then, the final reduced states for Alice’s and Bob’s quantum subsystems are given by

(49) $$\begin{array}{ccc} \rho_{A}^{\prime } & = & \frac{1}{N}\sum_{k=0}^{N-1}\rho_{A|B}^{k}=\frac{1}{N}1_{N}, \end{array}$$

(50) $$\begin{array}{ccc} \rho_{B}^{\prime } & = & \frac{1}{N}\sum_{k=0}^{N-1}{|k\rangle }_{B}\langle k|=\frac{1}{N}1_{N}. \end{array}$$

Therefore, the final reduced state for Alice’s subsystem remains unchanged, i.e., $\rho_{A}^{\prime }=\rho_{A}$. It is thus convenient to use Equation (48), as follows:

(51) $$\rho_{AB}^{\prime }=\frac{1}{N}\sum_{k,j=0}^{N-1}P_{kj}{|j\rangle }_{A}\langle j|⊗{|k\rangle }_{B}\langle k|.$$

From Equation (51), we can notice that, if Bob successfully discriminates the state $|\psi_{j}\rangle_{B}$, which is one of the states sent by Alice, the final shared state between Alice and Bob is ${|j\rangle }_{A}⊗{|j\rangle }_{B}$, and this occurs with probability $P_{jj}=P_{s}$. Otherwise, if the discrimination attempt fails, the resulting state is ${|j\rangle }_{A}⊗{|k\rangle }_{B}$ with k≠j, indicating an error in identifying the state $|\psi_{j}\rangle$. Such an error occurs with the minimum error probability, given by $P_{e}=1-P_{s}$.

We note that the minimum error discrimination of equidistant states does not require the use of sequential state discrimination protocols [53,54]. This is due to the fact that, within the ME strategy, the optimal measurement can be implemented as a single measurement that simultaneously minimizes the average probability of error. Consequently, the analysis can be carried out without invoking adaptive or sequential procedures, which are typically necessary in other discrimination strategies such as unambiguous discrimination [54].

## 6. Classical Correlations and Quantum Discord

In a bipartite quantum state $\rho_{AB}$, the total amount of correlation, in the many-copy scenario [55], is quantified by the quantum mutual information. This is defined as [55,56]

(52) $$I\left(\rho_{AB}\right)=S\left(\rho_{A}\right)+S\left(\rho_{B}\right)-S\left(\rho_{AB}\right),$$

where S(ρ) denotes the von Neumann entropy of the state ρ. In our ME scheme, we assume that Alice emits many independent and identically distributed (i.i.d.) copies of the bipartite state, i.e., $\sigma =\rho_{AB}^{n}$ for large *n* [57]. The entropy of the initial joint state $\rho_{AB}$, given by Equation (42), can be written as

(53) $$S\left(\rho_{AB}\right)=S\left(\rho_{A}\right)+\frac{1}{N}\sum_{j=0}^{N-1}S{(|\psi_{j}\rangle }_{B}\langle \psi_{j}|)=S\left(\rho_{A}\right),$$

since each $|\psi_{j}\rangle_{B}$ is a pure state and therefore has zero entropy. Thus, the total correlation between Alice and Bob in the bipartite state $\rho_{AB}$, as defined in Equation (45), is given by the mutual information $I\left(\rho_{AB}\right)=S\left(\rho_{B}\right)$. Moreover, the quantum mutual information, $I(\rho_{AB})$, can be written as [56,58]

(54) $$I\left(\rho_{AB}\right)=J\left(A|\left\{\Pi_{B}\right\}\right)+D\left(A|\left\{\Pi_{B}\right\}\right),$$

where $J(A|\left\{\Pi_{B}\right\})$ denotes the classical correlations and $D(A|\left\{\Pi_{B}\right\})$ the quantum discord. Both quantities depend on the measurement implemented by Bob, represented by the set of projectors $\left\{\Pi_{B}\right\}$. However, their sum, the total mutual information, is independent of the choice of measurement [59], i.e., they are complementary to each other [14]. The classical correlations $J(A|\left\{\Pi_{B}\right\})$ between Alice and Bob are defined as [58,60]

(55) $$J\left(A|\left\{\Pi_{B}\right\}\right)=S\left(\rho_{A}\right)-\frac{1}{N}\sum_{i=0}^{N-1}S\left(\rho_{A|b}^{i}\right),$$

which can be interpreted as the information about Alice’s system that Bob gains through the measurement $\left\{\Pi_{B}\right\}$. In this work, we focus on quantifying the classical correlations between Alice and Bob, $J(A|\left\{\Pi_{B}\right\})$, when Bob implements the ME on the set of pure equidistant states. On the other hand, the problem of maximizing the classical correlation $J(A|\left\{\Pi_{b}\right\})$ over all possible measurements performed by Bob is a challenging task and is not addressed here. The maximal classical correlation

(56) $$J\left(A|B\right)=\max_{\left\{\Pi_{B}\right\}}J\left(A|\left\{\Pi_{B}\right\}\right),$$

is known as the accessible information [9,10]. It corresponds to the classical mutual information maximized over all possible measurement strategies [12]. This optimization is generally difficult to perform and lies beyond the scope of the present work. Nevertheless, it is known that for N=2 the quantum measurement that achieves the accessible information coincide with the one that minimizes the error probability in state discrimination [4,11]. Furthermore, for any *N*, the accessible information for a set of *N* pure, nonorthogonal equidistant states must be at least as large as the classical information obtained through the ME measurement. The classical correlations $J(A|\left\{\Pi_{B}\right\})$ in ME, given the symmetry, can be expressed as

(57) $$J\left(A|\left\{\Pi_{B}\right\}\right)=S\left(\rho_{A}\right)-S\left(\rho_{A|B}^{0}\right),$$

where $S\left(\rho_{A}\right)=\log_{2}N$, and the entropy of the conditional state $\rho_{A|B}^{0}$ is given by

(58) $$S\left(\rho_{A|B}^{0}\right)=-\sum_{j=0}^{N-1}P_{0j}\log_{2}P_{0j},$$

where $P_{0j}$ is defined in Equation (47). On the other hand, quantum discord $D(A|\left\{\Pi_{B}\right\})$, which quantifies the quantum correlations consumed or lost during the measurement process, is given by

(59) $$D\left(A|\left\{\Pi_{B}\right\}\right)=S\left(\rho_{B}\right)-S\left(\rho_{A}\right)+S\left(\rho_{A|B}^{0}\right).$$

In our case, the quantum discord can be directly evaluated using Equation (59). Nevertheless, even for the relatively simple class of two-qubit *X* states, obtaining an analytical expression for quantum discord can be a challenging task, as it typically involves a nontrivial optimization over local measurements, as discussed in Refs. [61,62].

**Figure 6:**
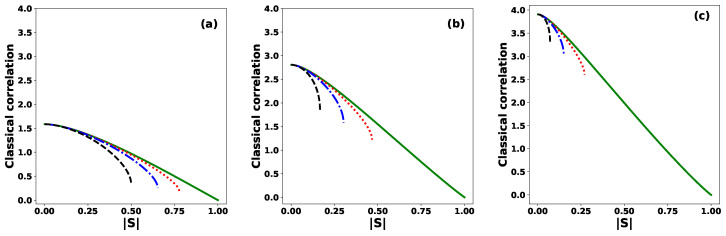
Classical correlation between Alice and Bob as a function of the modulus |S| of the inner product, for different values of the phase: θ=0 (solid green line), θ=π/6 (dotted red line), θ=π/3 (dashed–dotted blue line), and θ=π (dashed black line), for (**a**) N=3, (**b**) N=7, and (**c**) N=15.

Figure 6 shows the classical correlation between Alice and Bob as a function of the modulus |S| for (a) N=3, (b) N=7, and (c) N=15, and several values of the phase θ. The classical correlation reaches its maximum value when the equidistant states are orthogonal, i.e., |S|=0. In this case, the maximum value is $\log_{2}N$, indicating that Alice and Bob share total classical correlation. The corresponding final joint state is

(60) $$\rho_{AB}^{\prime }=\frac{1}{N}\sum_{j=0}^{N-1}{|j\rangle }_{A}\langle j|⊗{|j\rangle }_{B}\langle j|.$$

As |S| increases, the classical correlation $J(A|\left\{\Pi_{B}\right\})$ decreases, reaching its minimum value of zero when |S|=1 and θ=0. This behavior arises because the initial joint state between Alice and Bob becomes a product state with no correlation, given by

(61) $$\rho_{AB}=\left(\frac{1}{N}\sum_{j=0}^{N-1}{|j\rangle }_{A}\langle j|\right)⊗{|0\rangle }_{B}\langle 0|.$$

For a fixed value of *N* and a value of |S|, increasing the phase θ from 0 to π leads to a decrease in the classical correlation. Moreover, we observe that larger values of *N* result in higher classical correlation between Alice and Bob. This highlights the importance of using a greater number of states in order to enhance the classical correlation, which is a desirable feature for the performance of the protocol.

**Figure 7:**
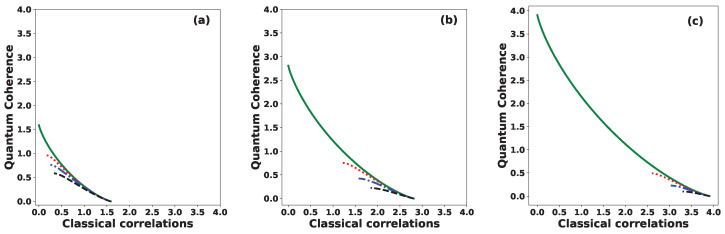
Quantum coherence versus classical correlation between Alice and Bob for different values of the phase: θ=0 (solid green line), θ=π/6 (dotted red line), θ=π/3 (dashed–dotted blue line), and θ=π (dashed black line), for (**a**) N=3, (**b**) N=7, and (**c**) N=15.

Figure 7 shows quantum coherence versus classical correlation between Alice and Bob for various values of the phase: θ=0 (solid green line), θ=π/6 (dotted red line), θ=π/3 (dashed–dotted blue line), and θ=π (dashed black line), for (a) N=3, (b) N=7, and (c) N=15. We observe that both quantities, quantum coherence and classical correlations, lie within the same range, namely $\left[0,\log_{2}N\right]$. It is important to highlight here the operational interpretation of the quantum coherence as a quantifier of cryptographic randomness gain [50,51]. Therefore, for a secure and efficient protocol, we expect that Alice and Bob share a significant amount of classical correlation while maintaining sufficiently high quantum coherence. For example, when the set of states is orthogonal |S|=0, the initial joint state takes the form $\rho_{AB}=\frac{1}{N}\sum_{j}{|j\rangle }_{A}\langle j|⊗|u_{j}\rangle_{B}\langle u_{j}|$, which exhibits a maximal classical correlation of $\log_{2}N$ bit but zero quantum coherence. In contrast, when the set of states is identical |S|=1, the initial joint state becomes $\rho_{AB}=(\frac{1}{N}\sum_{j}{|j\rangle }_{A}\langle j|)⊗{|0\rangle }_{B}\langle 0|$, a product state with no classical correlation but exhibiting maximal quantum coherence, equivalent to $\log_{2}N$ bits of randomness. Hence, Alice and Bob should choose an initial joint state $\rho_{AB}$ that simultaneously exhibits high classical correlation and high quantum coherence. This trade-off can be optimally achieved when the phase of the inner product is θ=0 and the number of states *N* is large.

## 7. Conclusions

We have studied the problem of minimum error discrimination for a set of *N* pure, nonorthogonal equidistant states, each prepared with equal a priori probability. The equidistant states resemble symmetric states but are uniquely characterized by a single complex parameter, which is the inner product *S* between any pair of the equidistant states. This feature allows for a detailed analysis of any quantity of interest as a function of the number of states in the set *N*, the modulus |S|, and the phase θ of the inner product. In this work, we described the behavior of the success probability, quantum coherence, and classical correlations under the minimum error strategy in terms of these parameters. We also proposed an experimental scheme for implementing minimum error discrimination of equidistant states. As an application, we present a quantum communication protocol in which Alice prepares and sends an equidistant state to Bob, who then applies the ME to extract the encoded classical information. This framework allows us to quantify the classical information shared between Alice and Bob through the ME process. Moreover, we determine the quantum coherence involved in Bob’s implementation of the ME, interpreting it as the gain of cryptographic randomness. Interestingly, our findings reveal a trade-off: greater classical information sharing between Alice and Bob corresponds to lower randomness generation in the protocol, and vice versa. Future work will focus on determining the accessible information, i.e., the maximal classical correlation achievable over all possible measurements, for the set of equidistant states. Although this quantity remains unknown, it must be no less than the classical information obtained under the ME. Moreover, in the special case of two pure nonorthogonal states, it is well established that the measurement that allows the extraction of the accessible information coincides with the minimum error measurement. Another perspective involves studying quantum coherence and classical correlations in more complex types of quantum states, such as Bell-diagonal states [63], which are mixtures of maximally entangled states and possess rich symmetry properties, or Bell-like states [64], which generalize Bell states to broader classes with controlled entanglement and coherence features. These families of states provide insightful frameworks for analyzing the interplay between different quantum resources under realistic noise and measurement settings.

## Data Availability

The original contributions presented in this study are included in the article. Further inquiries can be directed to the corresponding author.
